# Dual RNA-Sequencing Analysis of Resistant (*Pinus pinea*) and Susceptible (*Pinus radiata*) Hosts during *Fusarium circinatum* Challenge

**DOI:** 10.3390/ijms22105231

**Published:** 2021-05-15

**Authors:** Cristina Zamora-Ballesteros, Gloria Pinto, Joana Amaral, Luis Valledor, Artur Alves, Julio J. Diez, Jorge Martín-García

**Affiliations:** 1 Sustainable Forest Management Research Institute, University of Valladolid—INIA, 34004 Palencia, Spain; juliojavier.diez@uva.es (J.J.D.); jorgemg@pvs.uva.es (J.M.-G.); 2 Department of Vegetal Production and Forest Resources, University of Valladolid, 34004 Palencia, Spain; 3 Centre for Environmental and Marine Studies, CESAM, Department of Biology, University of Aveiro, 3810-193 Aveiro, Portugal; gpinto@ua.pt (G.P.); jsamaral@ua.pt (J.A.); artur.alves@ua.pt (A.A.); 4 Department of Organisms and Systems Biology, University of Oviedo, 33071 Oviedo, Spain; valledorluis@uniovi.es

**Keywords:** dual RNA-Seq, *Fusarium circinatum*, *Pinus pinea*, *Pinus radiata*, host-pathogen interaction, conifer defense, disease differential susceptibility

## Abstract

*Fusarium circinatum* causes one of the most important diseases of conifers worldwide, the pine pitch canker (PPC). However, no effective field intervention measures aiming to control or eradicate PPC are available. Due to the variation in host genetic resistance, the development of resistant varieties is postulated as a viable and promising strategy. By using an integrated approach, this study aimed to identify differences in the molecular responses and physiological traits of the highly susceptible *Pinus radiata* and the highly resistant *Pinus pinea* to *F. circinatum* at an early stage of infection. Dual RNA-Seq analysis also allowed to evaluate pathogen behavior when infecting each pine species. No significant changes in the physiological analysis were found upon pathogen infection, although transcriptional reprogramming was observed mainly in the resistant species. The transcriptome profiling of *P. pinea* revealed an early perception of the pathogen infection together with a strong and coordinated defense activation through the reinforcement and lignification of the cell wall, the antioxidant activity, the induction of PR genes, and the biosynthesis of defense hormones. On the contrary, *P. radiata* had a weaker response, possibly due to impaired perception of the fungal infection that led to a reduced downstream defense signaling. *Fusarium circinatum* showed a different transcriptomic profile depending on the pine species being infected. While in *P. pinea,* the pathogen focused on the degradation of plant cell walls, active uptake of the plant nutrients was showed in *P. radiata*. These findings present useful knowledge for the development of breeding programs to manage PPC.

## 1. Introduction

*Pinus radiata* is the world’s most widely planted tree for its economic value [1]. In the last two centuries, its fast growth and wood quality have satisfied the increasing demand for timber and wood products worldwide. In the North of Spain, there are large areas of *P. radiata* plantations (260,000 ha) where this species represents the first conifer species in volume harvested (5 million of m^3^) [2]. Unfortunately, the Spanish Atlantic region has witnessed a proliferation of fungal diseases that have severely affected the plantations of *P. radiata* in the last decades. This conifer is especially susceptible to diseases such as Dothistroma needle blight (DNB), brown spot needle blight (BSNB), or pine pitch canker (PPC), which have led to an important economic impact [3,4].

The invasive fungus *Fusarium circinatum* is the causal agent of the PPC, being considered one of the most important pathogens of conifers globally [5]. Infected seeds are the main pathway of introduction in nurseries, where the pathogen causes pre- and post-emergence damping-off and mortality of seedlings [6,7]. In the field, infected trees suffer from stem cankers, dieback, and even branches and trunks girdling that cause tree death [8]. The global distribution of *F. circinatum* is mainly due to globalization in terms of international trade of plant materials and the failed current regulation [9,10,11]. Recently, it has been discovered that *F. circinatum* has the ability to endophytically colonizing pine but also non-coniferous species [12,13]. This fact, besides making the control of the disease especially challenging, highlights the importance of using scientific advances in the development of effective strategies [11].

Once established, no viable intervention measure aiming at the control or eradication of PPC is available. In this context, reforestation of the damaged areas with genetically resistant material seems to be one of the most promising strategies to reduce the impact of this disease [14]. Although *P. radiata* is the most susceptible species of PPC, *F. circinatum* can infect up to 60 different species of *Pinus*, *Pseudotsuga menziesii,* and species in genera *Picea* and *Larix* [4,15,16]. Among the host species, there is a wide range of susceptibility variation to *F. circinatum* [6,17], being the native Mediterranean *Pinus pinea* one of the most resistant [18,19]. Recently, under an intergovernmental framework for combating PPC (European COST action FP1406), the susceptibility level of provenances of several European conifers pointed out that some interspecific genetic resistance could be found [7,16,20]. Intraspecific resistance, which has been related to environmental gradients or adaptive processes, has been used for successful breeding programs [21]. In addition, hybridization with resistant species or populations showed suitable results [22,23]. In Spain, due to the ban on planting susceptible species in infected areas (Spanish Royal Decree 637/2006 and 65/2010), the use of alternative species such as eucalyptus is already a fact. In this context, further omics studies are required to understand the mechanisms underlying disease overcoming and to achieve resistant genotypes based on this knowledge.

Conifer trees have several defense mechanisms that protect them against pathogens. The defensive system of pines includes mechanical barriers such as the induced activation of traumatic resin ducts and cell wall reinforcement and chemical defenses, including the production of oleoresin terpenoids [24]. The allocation of resources must be regulated by the tree in order to maintain a balance between growth and defense, which is key to tree resistance [25]. In turn, fungal pathogens have developed sophisticated penetration, infection, and colonization strategies to overcome and suppress these defense mechanisms [26]. In the last decade, an increased number of studies have examined the pathosystems of several forest trees at the transcriptional level [27,28,29,30,31,32]. The development of NGS and associated bioinformatics pipelines have made feasible the application of high-throughput transcriptomic analysis to unsequenced species, allowing a deep description of the transcriptional mechanisms implied in plant fungal defense [33,34,35,36,37]. Specifically, some studies have examined the response of different pine species to *F. circinatum*. These included a comparative transcriptomic analysis between two contrasting genotypes of *P. radiata* inoculated by *F. circinatum*, where differential expression of several pathogenesis-related (PR) genes, phosphorylase family (PFP), and non-race specific disease resistance (NDR1) genes were found associated with the resistant genotype [38]. Moreover, some important defense-related genes such as phenylalanine ammonia-lyase (PAL) were down-regulated in the highly susceptible *Pinus patula* at a very early stage of infection (after 24 h) [39]. Visser et al. [40] focused their study only on PR genes in both *P. patula* and the resistant host *P. tecunumanii* after *F. circinatum* infection. However, none of these studies examined the transcriptome of the pathogenic fungus upon infection.

Current sequencing platforms generate an unprecedented number of reads, making possible the simultaneous analysis of transcripts both of pathogens and their hosts [41]. This approach, called dual RNA-Seq, has been employed to reveal the interaction of *Pinus pinaster* with *F. circinatum* [42]. The moderate resistance of *P. pinaster* was reflected in the early induction of genes associated with a complex phytohormone signaling of jasmonic acid (JA), ethylene (ET), and salicylic acid (SA). On the other hand, it was suggested a perturbation in the host phytohormone homeostasis by *F. circinatum* through the expression of fungal genes related to these phytohormones [42]. Plant hormones are important regulators of metabolism being closely related to the plant defense [43], resulting in a complex signaling cross-talk of phytohormones such as JA, ET, SA, auxins, cytokinins (CKs), gibberellins (GAs), abscisic acid (ABA), and brassinosteroids (BRs). JA and ET are involved in the activation of defense responses against necrotrophic pathogens, while biotrophic and hemibiotrophic (as *F. circinatum*) pathogens are generally associated with SA induction and the establishment of systemic acquired resistance (SAR) [44]. SA and JA/ET defense pathways are in general mutually antagonistic, although synergistic interactions have been reported as well [44]. Recently, Visser et al. [45] focused their transcriptomic study on the hormone regulation in *P. patula* and *P. tecunumanii* challenged with *F. circinatum*. In this study, a stronger and earlier response was observed in the resistant *P. tecunumanii* through the coordination of auxin, ET, JA, and SA signaling*,* whereas *P. patula* showed a delayed response, which was associated with its susceptibility to *F. circinatum*. In addition, the expression of genes related to ergosterol biosynthesis in *F. circinatum* was lower during the infection of the resistant host, which was pointed to a higher vulnerability of the pathogen to conifer PR1 genes. Physiological and hormonal changes have also been investigated in the *Pinus*-*F. circinatum* interaction. While symptomatic *P. radiata* and *P. pinaster* suffered a water deprivation-like scenario and photosynthetic limitation after *F. circinatum* infection, *P. pinea* opened their stomata, incrementing the transpiration and accumulated glycerol [19]. Furthermore, Amaral et al. [46] suggested that ABA catabolism could play a key role in PPC overcome.

Gene activation and transcription, protein and enzyme activities, primary and secondary metabolism, and hormones and metabolites signaling are the main mechanisms reprogrammed in plants under pathogen challenges. To understand the processes that underlie the resistance of the Mediterranean species *P. pinea* and the susceptibility of the typically allochthonous species *P. radiata* against the pathogenic fungus *F. circinatum*, a comprehensive study of the regulation of the array of genes involved in pine defense mechanisms has been performed. Physiological analyses were carried out in order to further support the results obtained at the transcriptional level. This combined transcriptomic and physiological approach is expected to improve the understanding of host-pathogen interaction and identify key players and pathways that may be useful for the development of resistant genotypes through breeding and/or genetic engineering.

## 2. Results

### 2.1. Differences in the Susceptibility Between the Hosts

**Figure 1:**
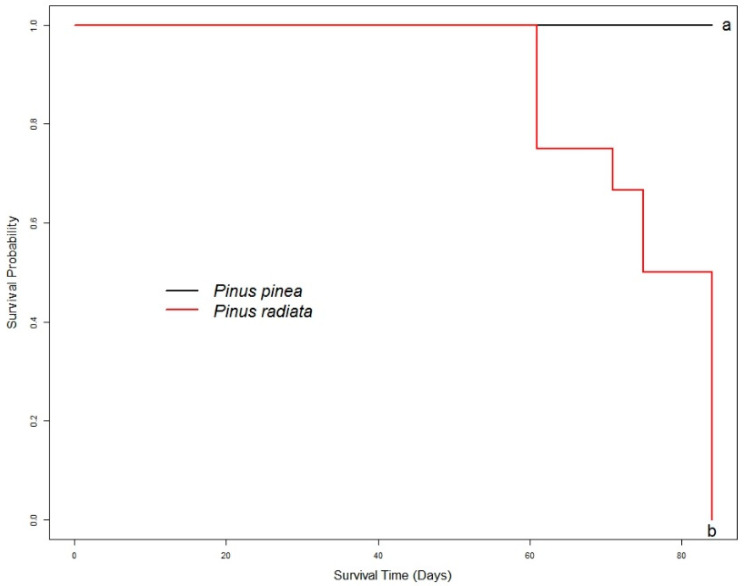
Survival probability plot determined using the Kaplan–Meier estimate of the survival function for *P. pinea* and *P. radiata* seedlings inoculated with *F. circinatum*. Different letters indicate significant p-values.

At the time of sampling, none of the seedlings showed symptoms of the disease. In order to validate the susceptibility of the hosts, 12 seedlings were monitored for each species during the 84 days. Inoculated seedlings of *P. radiata* began to die 61 days after inoculation and, by the end of the experiment, all of them had died (Figure 1). On the contrary, no mortality was recorded for inoculated *P. pinea* seedlings. In addition, inoculated *P. pinea* seedlings did not show symptoms of the disease by the end of the experiment, indicating an overcoming of the disease. Statistically, the survival analysis revealed significant differences between both species (χ^2^ = 22.7, *p* < 0.001).

### 2.2. Physiological Measurements

**Figure 2:**
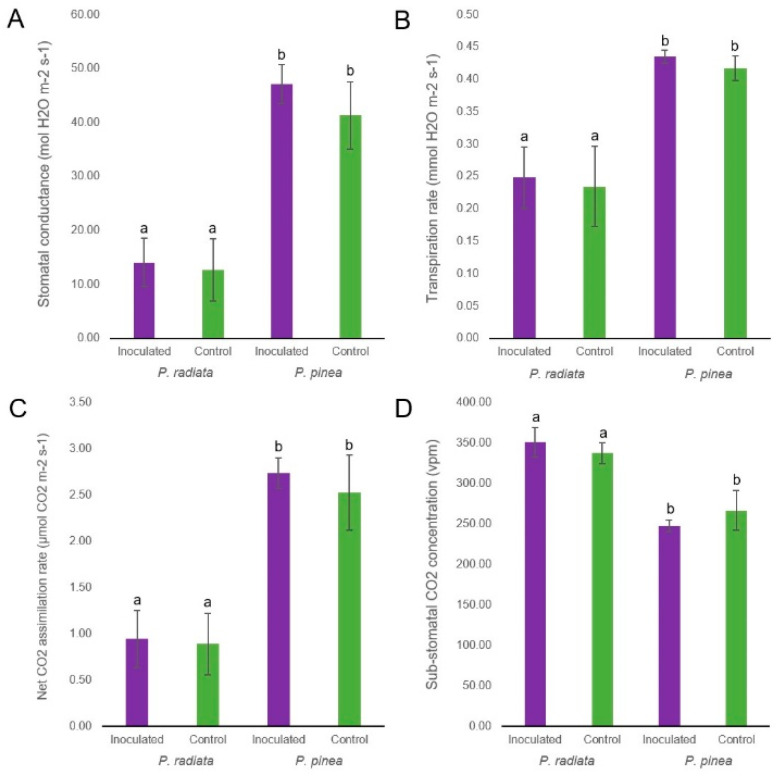
Needle gas exchange-related parameters of *P. pinea* and *P. radiata* inoculated with *F. circinatum* and controls at 4 dpi. (**A**) Stomatal conductance. (**B**) Transpiration rate. (**C**) Net CO_2_ assimilation rate. (**D**) Sub-stomatal CO_2_ concentration. Error bars show the standard deviation. Different letters above the bars indicate significant differences (ANOVA, *p* < 0.05).

The water potential of both species was not significantly affected by fungal infection, although a slight reduction in *P. radiata* values and an increase in *P. pinea* can be observed (Figure S1). The analysis confirmed that water potential is significantly influenced by the species. Similarly, the gas exchange and stomatal conductance parameters were only significantly different between species but not influenced by the pathogen effect (Figure 2). The net CO_2_ assimilation rate (A), the stomatal conductance (gs), and the transpiration rate (E) were higher in *P. pinea*, while the needle CO_2_ concentration (Ci) was slightly higher in *P. radiata*.

### 2.3. Dual RNA-Sequencing Statistics

**Figure 3:**
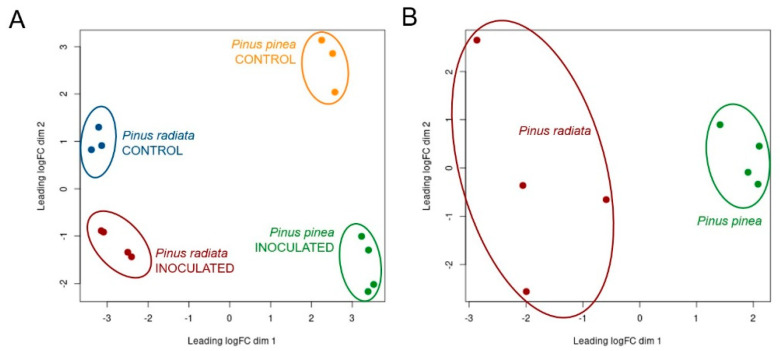
Two-dimensional scatterplot of the principal component analyses (PCA) for (**A**) *Pinus radiata* and *P. pinea* different treatments and (**B**) *Fusarium circinatum* infecting every host species. The distances approximate the typical log_2_ fold changes between the samples.

The characterization and comparison of *P. pinea* and *P. radiata* responses to *F. circinatum* infection and the simultaneous characterization of the *F. circinatum* gene expression were carried out using a dual RNA-Seq approach. An average of 51.8 ± 4.41% of *P. pinea* reads, and 82.9 ± 1.84% of *P. radiata* reads were mapped to the reference genome of *P. taeda* (Table S1). Considering the infected samples, for *P. pinea* and *P. radiata*, respectively, an average of 1.86 ± 2.33% and 0.57 ± 0.29% reads mapped to the *F. circinatum* genome. The PCA allowed identifying two sample outliers in the control treatments of each pine species that were discarded from the downstream analysis (Figure S2). Once the atypical samples were removed, the first principal component in the host analysis showed a strong separation by pine species. On the second principal component, the different treatments (infected by *F. circinatum* and control) were clustered in separated groups, being more evident between the *P. pinea* treatments (Figure 3A). The visualization of the *F. circinatum* expression analysis with PCA showed a clear separation according to pine species infected (Figure 3B).

**Figure 4:**
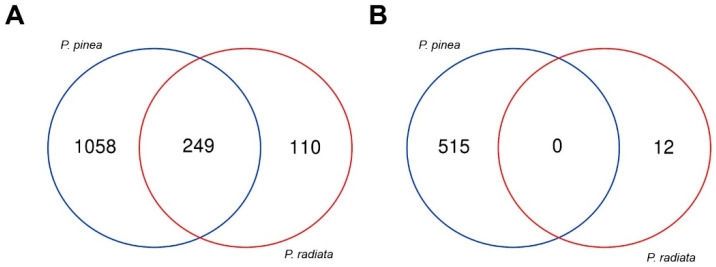
Venn diagram showing the number of *P. pinea* and *P. radiata* genes up-regulated (**A**) and down-regulated (**B**).

For the host dataset, transcripts with counts per million (CPM) > 1 in at least three libraries (the minimum number of libraries among treatments) were considered reasonable to use in further comparisons. In the case of the pathogen dataset, only genes present in at least four replicates with CPM > 2 were used for DE analysis. A total of 20,380 genes were above abundance and consistency between replicates thresholds in the hosts and 8866 genes in the pathogen. DE analysis of *P. pinea* (inoculated vs. control) identified 1822 DEGs, whereas 371 significant DEGs were identified for *P. radiata* (Table 1; Tables S2 and S3; Figure S3). Only 249 up-regulated DEGs out of 1417 were shared in the defense response between both species, without sharing any of the repressed genes (Figure 4). *Fusarium circinatum* DE analysis of inoculated samples (*P. pinea* vs. *P. radiata*) identified 204 significant DEGs (Table 1; Table S4; Figure S4).

The results of the combined annotation of *F. circinatum* through the EnTAP pipeline yielded 9757 transcripts with coding regions predicted using GeneMarkS-T (Table S5). The best-hit selection of BLASTp, which represents the most informative match available, combining the hits from each database within the coverage and e-value thresholds specified, resulted in alignments for 9406 (78%) proteins. The annotated transcripts were mostly shared with *Fusarium* species. EggNOG annotation assigned 9527 (97%) *F. circinatum* sequences to families, and InterProScan annotation assigned 6803 (70%) to domains. To sum up, a total of 9650 (99%) sequences were annotated, of which 7434 (76%) and 2494 (26%) were assigned gene ontology (GO) terms and KEGG pathways, respectively. To identify putative genes involved in pathogenicity, we analyzed the *F. circinatum* sequences for similarity with subjects in the pathogen-host interaction gene database (PHI-base), resulting in 2489 (26%) alignments (Table S6).

### 2.4. Over-Represented Gene Ontologies and KEGG Pathways in Host Datasets Challenged With Fusarium circinatum

**Figure 5:**
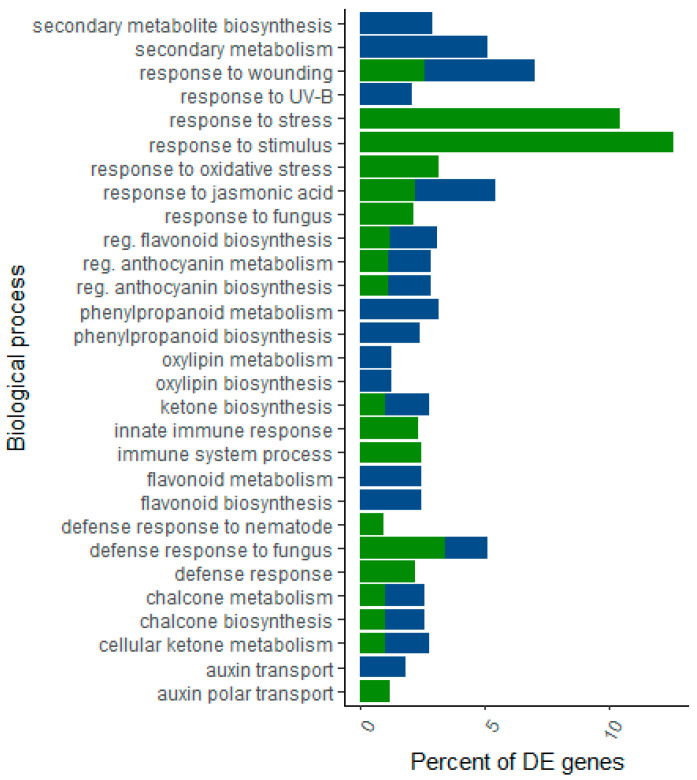
Representation of the most significantly (*p* < 0.05) enriched GO terms (biological processes) of the up-regulated genes of *P. pinea* (blue bars) and *P. radiata* (green bars) infected by *F. circinatum*.

The DEGs in the host datasets were analyzed to identify over-representation of GO terms focusing on biological processes (BP) and molecular function (MF), as well as of KEGG pathways (*p*-value < 0.05; Tables S7–S10). This analysis has shown the wide range of biological processes underlying each host species in response to *F. circinatum*. The up and down-regulated gene datasets of *P. pinea* shared the majority of enriched GO terms and were related to plant defense processes such as oxidation-reduction, flavonoid biosynthesis and metabolism, ethylene, and jasmonic acid biosynthesis, regulation of anthocyanin metabolism, and oxylipin biosynthesis. No GO terms or KEGG pathways were enriched in the down-regulated dataset for *P. radiata* DEGs, but a large number of GO terms were enriched in the up-regulated DEGs. Most of them were also found to be enriched in the *P. pinea* datasets (Figure 5).

The majority of enriched KEGG pathways were found in both up- and down-regulated datasets of *P. pinea*. The pathways uniquely enriched in the up-regulated genes were related to plant defense, such as plant hormone signal transduction, zeatin biosynthesis, L-ascorbate oxidase activity, and ubiquinone and other terpenoid-quinone biosynthesis; contrasting with the exclusive down-regulation of genes related to the energy metabolism of the plant. The most enriched KEGG pathways for the up-regulated DEGs in *P. radiata* were secondary metabolites biosynthesis and plants circadian rhythm pathway. Over-represented datasets of both hosts shared most of the KEGG pathways as well, although there were more pathways unique to the resistant host than to the susceptible species.

### 2.5. Expression of Genes Involved in Pinus Response to Fusarium circinatum Infection

The infection by *F. circinatum* has caused severe transcriptional reprogramming in both pine species. Several of these genes belong to functional groups involved in plant defense against pathogens, such as signal perception and transcriptional regulation, secondary metabolism, antimicrobial activity, cell wall reinforcement and lignification, and pathogenesis-related genes. A closer look at the DEGs in each host species revealed some differences in response to *F. circinatum* regarding these processes (Table S11), which could be key for pine resistance to PPC.

**Figure 6:**
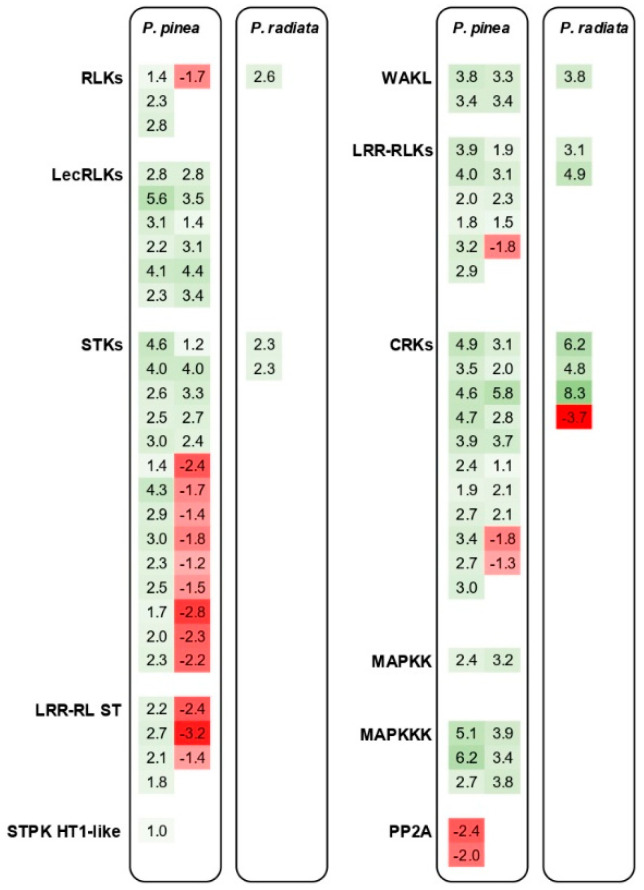
Differentially expressed genes (DEGs) with a role in signal perception. Numbers represent the log_2_ fold change value based on the comparison of the transcript levels between the pine seedlings infected by *F. circinatum* and mock-inoculated control. Groups of genes are abbreviated as follows: RLKs, receptor-like kinases; LecRLKs, lectin domain-containing receptor kinase; STKs, serine/threonine-protein kinases; LRR-RL ST, receptor-like serine/threonine-protein kinase with leucine-rich repeat domain; STPK HT1-like, serine threonine-protein kinase HT1-like; WAKL, wall-associated receptor kinase-like; LRR-RLKs, leucine-rich repeat receptor-like protein kinases; CRKs, cysteine-rich receptor-like protein kinase; MAPKK, mitogen-activated protein kinase kinase; MAPKKK, mitogen-activated protein kinase kinase kinase; PP2A, serine/threonine phosphatases type 2A.

Belonging to the pattern recognition receptor (PRR) family, different classes of receptor kinases such as leucine-rich repeat (LRR) receptor-like kinases (RLKs), serine/threonine-protein kinases (STKs), and cysteine-rich receptor-like protein kinases (CRKs) were highly abundant among the DEG in *P. pinea*; while only seven were up-regulated in *P. radiata* (Figure 6). Several wall-associated receptor kinase-like (WAKL) genes were mainly up-regulated in *P. pinea*, whereas only one did in *P. radiata*. Furthermore, 12 genes encoding lectin domain-containing receptor kinase proteins, which have a role in chitin-containing pathogen recognition, were exclusively up-regulated in the resistant species (Figure 6). As a protective mechanism, two serine/threonine phosphatases type 2A (PP2A) were down-regulated in *P. pinea*. PRR-derived signals trigger mitogen-activated protein kinases (MAPK) cascades and calcium-dependent protein kinases (CDPKs). Several mitogen-activated protein kinase kinase (MAPKK) and mitogen-activated protein kinase kinase kinase (MAPKKK) were up-regulated in *P. pinea* (Figure 6). Genes related to calcium (Ca^2+^) signaling, including calmodulin and calcineurin B–like (CBL) interacting protein kinases (CIPKs), were up and down-regulated in *P. pinea*. Particularly, two genes encoding CIPK3 protein kinases, which are implicated in stress and ABA responses, were up-regulated.

A large number of genes that encode R proteins were mainly up-regulated in *P. pinea*; while only one was up-regulated in the susceptible species and another one was highly down-regulated (|log_2_[Fold Change]| = 6.6). WRKY transcription factors (WRKY75, 72, 51, 14, 59, and 17) were induced in the DEGs of the resistant species. Only one WRKY75 was weakly up-regulated in *P. radiata*. An ethylene response factor (ERF) was expressed by both species, this time, to a greater extent in the *P. radiata*. Members of other families of transcription factors such as MYB and bZIP were up- and down-regulated in *P. pinea*.

**Figure 7:**
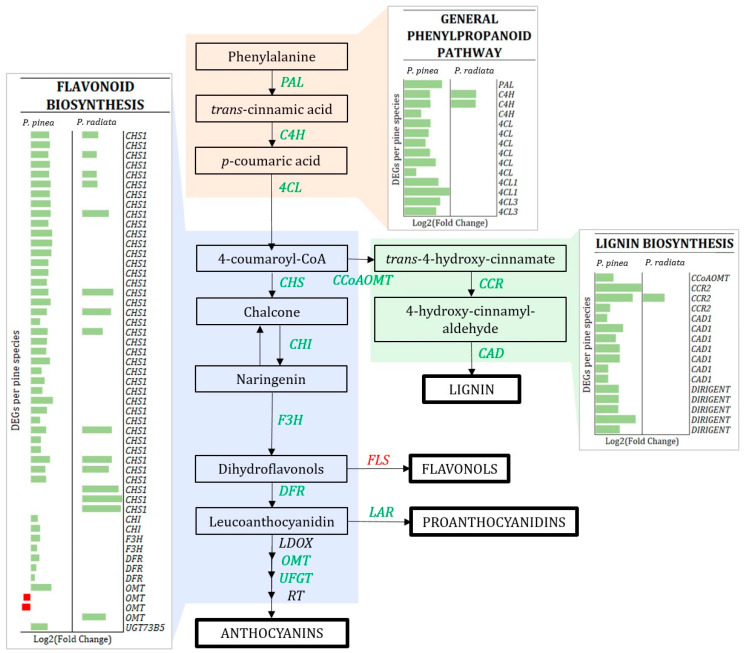
Simplified scheme of the flavonoid and lignin biosynthetic pathway. Some critical up-regulated (green) or down-regulated (red) enzymes of *P. pinea* are indicated and abbreviated as follows: *PAL*, phenylalanine ammonia-lyase; *C4H*, cinnamate 4-hydroxylase; *4CL*, 4-coumarate: CoA ligase; *CHS*, chalcone synthase; *CHI*, flavanone isomerase; *F3H*, Flavanone 3-hydroxylase; *DFR*, dihydroflavonol 4-reductase*; LDOX,* leucoanthocyanidin dioxygenase; *OMT*, *O*-methyltransferase; *UFGT*, UDP-glucose:flavonoid 3-*O*-glucosyl transferase; *RT*, putative rhamnosyltransferase; *CCoAOMT*, caffeoyl-CoA *O*-methyltransferase; *CCR*, cinnamoyl-CoA reductase; *CAD*, cinnamyl alcohol dehydrogenase; *FLS*, flavonol synthase; *LAR*, leucanthocyanidin reductase. Individual differentially expressed genes (DEGs) with annotated functions are listed along the Y-axis for *P. pinea* and *P. radiata*. The bars in the X-axis represent the level of log_2_ fold change value based on the comparison of the transcript levels between the pine seedlings infected by *F. circinatum* and mock-inoculated control. Red bars denote the level of down-regulation, and green bars indicate the level of up-regulation.

The production of secondary metabolites through the phenylpropanoid pathway is essential for plant defense [47] as it supplies precursors to lignin biosynthesis. Pine species exhibited a differential response of genes related to the biosynthesis of phenolic compounds (Table S11; Figure 7). This pathway was widely induced upon pathogen infection in *P. pinea*, and induction of genes encoding leucoanthocyanidin reductase (LAR) and alkaloid berberine proteins was observed in both species. Regarding isoflavonoids biosynthesis, several isoflavone reductases (IFRs) were up-regulated in *P. pinea*. A considerable number of predicted terpene synthases were up-regulated in *P. pinea,* with only half of them present in *P. radiata*, most of them uniquely in these samples. Other pathogenesis-related proteins with antifungal activity, such as cysteine-rich secretory proteins, were up-regulated in both species (log_2_[Fold Change] of 8 and 10).

**Figure 8:**
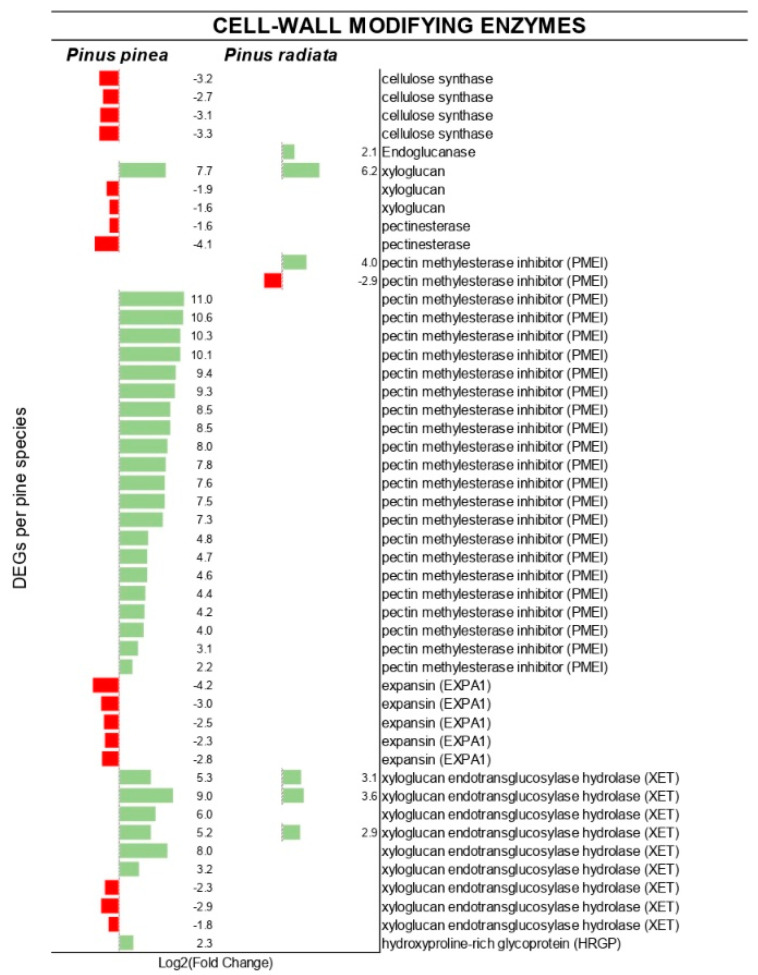
Differentially expressed genes (DEGs) encoding cell wall modifying family proteins. Individual genes are listed along the Y-axis. In X-axis is represented the log_2_ fold change value based on the comparison of the transcript levels between *F. circinatum* infected *Pinus* species and mock-inoculated plants.

The DE of genes encoding for cell wall modifying enzymes of both pines is shown in Figure 8. Interestingly, a contrasting scenario is shown in the pectinesterase (PE) and pectin methylesterase inhibitor (PMEI) genes: while 21 genes encoding for PMEI were highly induced (up to 10.9 log_2_[fold change]) in *P. pinea*, only one did in *P. radiata,* and another was repressed. Additionally, two PE were down-regulated only in *P. pinea*. Furthermore, many genes related to lignin biosynthesis were up-regulated in *P. pinea*, being this number much smaller in *P. radiata* (Figure 7).

The expression profile for DEGs encoding transporters demonstrated differences in infection response between host species. Genes encoding pleiotropic drug resistance (PDR)-type ABC transporters, multidrug resistance-associated proteins (MRP)-type ABC transporters, and other ABC transporters were up-regulated in *P. pinea*; in contrast, only one (PDR)-type ABC transporter was up-regulated in *P. radiata*. Similarly, several genes encoding chloroplast membrane-localized members of the multidrug and toxin (MATE) transporters were up-regulated in *P. pinea*, but only one in *P. radiata*. Other transporter families, including aluminum-activated malate, amino acid, ammonium, bidirectional sugar, lysine/histidine, nitrate, and potassium transporters, were also differentially regulated in *P. pinea* by *F. circinatum* infection. In *P. radiata*, very few of these transporters were expressed, being zinc transporters the most represented group with five genes highly induced. Interestingly, genes encoding for nodulin-like proteins, which are involved in the transport of different molecules, were far more induced in *P. radiata* than in *P. pinea*. Indeed, an early nodulin showed one of the highest fold changes (9.3) of the expression levels of *P. radiata*.

Protease inhibitors were one of the most outstanding classes of defense-related genes in the analysis. In total, 18 genes showing similarity to various types of protease inhibitors (Kunitz-type, potato type II serine, and cysteine proteinase inhibitors) were highly up-regulated after pathogen infection. From these, 12 were shared between both pine species, with *P. radiata* showing the highest fold changes. Genes encoding PR proteins were highly induced (up to 12 log_2_[fold change]) in both host species, although these genes were more abundant in the resistant species. Among these, one β-1,3-glucanase was up-regulated in *P. pinea*, as well as a considerable number of chitinases and thaumatin-like proteins in both species. A large number of peroxidases were up- and down-regulated in *P. pinea* and highly up-regulated in *P. radiata*. Twenty-nine genes encoding glutathione S-transferases (GST) involved in detoxification of toxic products triggered by the oxidative burst were up-regulated in *P. pinea*, whereas only five GSTs were induced in *P. radiata*. Other genes related to ROS detoxification through the ascorbate-glutathione pathway, such as L-ascorbate and bifunctional monodehydroascorbate reductase and carbonic anhydrase, were induced in the resistant species.

### 2.6. Differences in Hormone Signaling Pathways Between Pine Species

**Figure 9:**
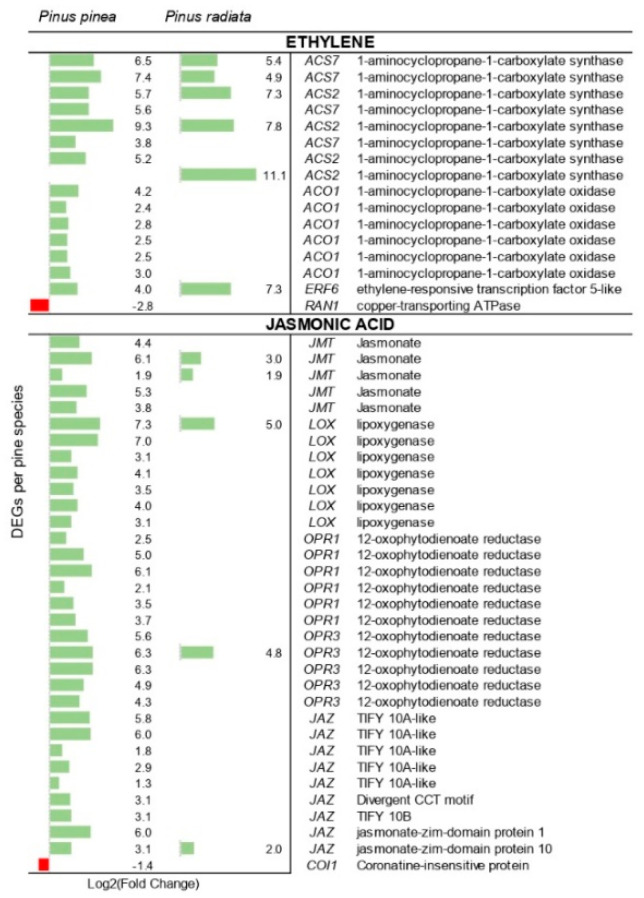
Differentially expressed genes (DEGs) encoding enzymes of defense hormone biosynthesis. Individual genes are listed along the Y-axis. In X-axis is represented the log_2_ fold change value based on the comparison of the transcript levels between *F. circinatum* infected *Pinus* species and mock-inoculated plants.

Several DEGs involved in phytohormone pathways were found in both the *P. pinea* and the *P. radiata* datasets (Table S12). Several genes coding for 1-aminocyclepropane-1-carboxylic acid (ACC) synthase (ACS), which catalyzes the first step of ET biosynthesis [48], were overexpressed in both pine species, although in greater number in *P. pinea* (Figure 9). The gene coding for the enzyme involved in the second step of the ET biosynthesis, the ACC oxidase (ACO) gene, was only induced in *P. pinea*. Moreover, the copper transporter RAN1 (responsive to antagonist 1), required for the biogenesis of the ET receptors, was down-regulated in the resistant species.

The number of genes involved in JA signaling was considerably higher in *P. pinea* than in *P. radiata* (Figure 9). While several genes encoded JA-metabolizing enzymes as jasmonate methyl transferase (JMT), and JA biosynthesis-related enzymes lipoxygenases (LOX) and 12-oxo-PDA-reductase (OPR) were up-regulated in inoculated *P. pinea*; only four of these were up-regulated in *P. radiata*. Although this could indicate activation of JA signaling, genes that encode jasmonate-zim-domain (JAZ) transcription repressors (TIFY domain-containing proteins) were also up-regulated in *P. pinea* and one in *P. radiata*; and a coronatine insensitive 1 (COI1) predicted gene, essential for the regulation of JA signaling [49], was down-regulated in *P. pinea*.

The product of the shikimate pathway chorismate is the initial metabolite in the synthesis of salicylic acid (SA). While in *P. pinea*, genes from the shikimate and phenylalanine ammonia-lyase (PAL) pathways were up-regulated, no DE of SA biosynthesis genes was observed in *P. radiata*. Several genes encoding phytoalexin deficient 4 (PAD4) lipase-like proteins were up-regulated in *P. pinea*, but two were down-regulated. The same number of genes belonging to the S-adenosyl-L-methionine carboxyl methyltransferase (SAMT) family protein was induced in both pine species, one of those genes being shared between them.

Four type 2C protein phosphatase (PP2C) genes involved in the inhibition of the abscisic acid (ABA) signaling transduction were up- and down-regulated in *P. pinea*. The down-regulation of an ABA receptor in *P. pinea* could indicate suppression of ABA signaling. However, the up-regulation of two protein kinases, CIPK20, contradicts this. A predicted ABA 8’-hydroxylase was exclusively identified in *P. radiata*. Additionally, a number of the positive regulator BG3 genes that encode β-glucosidases were highly up-regulated in both *P. pinea* and *P. radiata*. Interestingly, not one BG3 gene was common among species. In addition, 10 genes belonging to the abscisic acid stress ripening (ASR) gene family, which are induced by ABA and abiotic stress [50], were up-regulated in *P. pinea*, except one ASR1 that was down-regulated.

Genes related to the biosynthesis of indole-3-acetic acid (IAA), the main auxin in plants, were not present in the DEGs datasets. However, 15 genes encoding auxin-induced proteins were up-regulated in *P. pinea*. Several auxin-repressed genes and one auxin-responsive gene (Aux/IAA) were down-regulated. This could be related to the overexpression of several genes involved in the degradation of Aux/IAA by the ubiquitin pathway (Skp-Cullin-F-box [SCF] complex); genes encoding ubiquitin ligase (E3), ubiquitin-conjugating (E2) enzymes, and polyubiquitins; F-box-related genes and RING-finger genes. Genes related to the IAA inactivation pathways, including IAMT1 (IAA carboxyl methyltransferase 1) and GH3 (Gretchen Hagen 3 acyl acid amido synthetase family proteins) were also up-regulated in *P. pinea*. Additionally, auxin efflux carrier genes that play a crucial role in polar auxin transport were down-regulated in this species. In *P. radiata*, far fewer auxin-related DEGs were found to be up-regulated: several auxin-induced genes; and some genes of the Skp-Cullin-F-box (SCF) complex such as ubiquitin ligase (E3), RING-finger, F-box-related genes, and a cullin 1 (CUL1).

Brassinosteroid biosynthesis was represented neither in *P. pinea* nor in *P. radiata* DEGs. However, BR signaling-related genes DE in *P. pinea*: a BR receptor BRI1 (BR insensitive 1) gene and other signaling elements were down-regulated; a transcription factor of the family BRI1-EMS-suppressor 1 (BES1), brassinazole-resistant 1 (BZR1), and SERK1, a coreceptor of BRI1, were up-regulated. The transcription factor PIF3, which, along with BZR1, allows for BR-induced gene expression, was down-regulated. In contrast, genes involved in GA biosynthesis were identified in *P. pinea*. While one ent-kaurene oxidase (KO) was down-regulated, two putative ent-copalyl diphosphate synthase (CPS) were highly up-regulated. GA 20-oxidase (GA20OX) gene is required for the release of bioactive forms of gibberellins and was up-regulated in *P. pinea* (5.4 log2[fold change]). However, a GA 2-oxidase (GA2OX), which converts active GAs into their inactive form, was also up-regulated (2.7 log2[fold change]). This, together with the fact that a gibberellin-regulated protein (SN2) was down-regulated, could suggest suppression of GA activity. Moreover, enzymes involved in cytokinins (CKs) degradation was identified: up- and down-regulation of cytokinin oxidase/dehydrogenase (CKX) and UDP-glycosyl transferase (UGT) genes in *P. pinea*; and up-regulation of UGT genes in *P. radiata*.

### 2.7. Over-Represented Gene Ontologies in Fusarium circinatum Dataset

**Figure 10:**
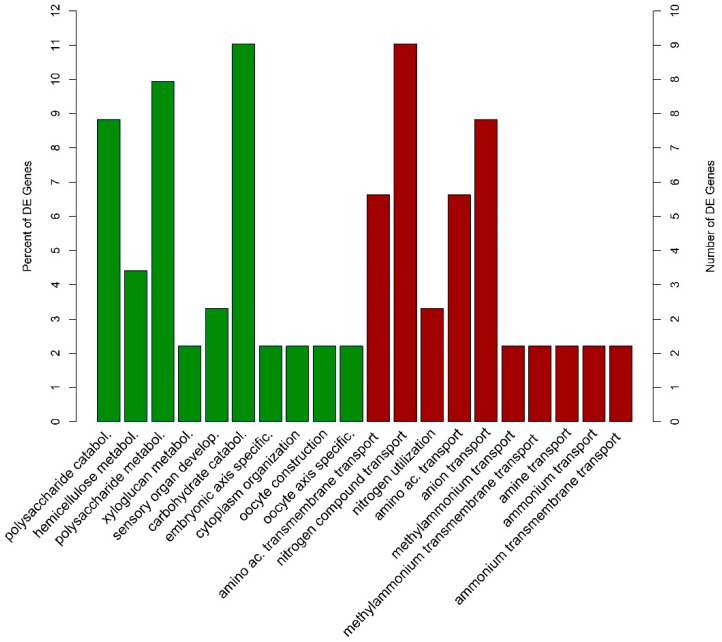
Representation of the 10 most significantly (*p* < 0.05) enriched GO terms (biological processes) of the up-regulated (green bars) and down-regulated (red bars) genes of *F. circinatum* infecting *P. pinea* compared with *P. radiata*.

The GO enrichment analysis of the DEGs in the pathogen dataset showed a strong difference in the fungal response depending on the pine species (Table S13). The genes were DE in *P. pinea* relative to *P. radiata*; namely, the up- and down-regulated genes were significantly expressed in *P. pinea* with respect to *P. radiata*. Only a few GO terms were shared between up- and down-regulated set of genes, including carboxylic acid, organic acid, and amino acid transport. In the up-regulated gene dataset, BP terms associated with degradation of amino acids and sugars/carbohydrates (e.g., hemicellulose, xyloglucan, arginine) and the developmental and cell process were enriched (Figure 10); as well as several terms related to ornithine metabolism. Interestingly, terms related to lignin and xylan degradation were uniquely enriched in the up-regulated dataset. Almost half of the BP terms in the down-regulated dataset were related to cellular transport (Figure 10). The oxidation-reduction process, chitin catabolism, benzoate catabolism, and several terms related to fungal growth were also over-represented in the down-regulated dataset.

Only three MF terms were present in both datasets, which were related to carboxylic acid and organic acid transmembrane transport. Cellulose binding, hydrolase activity, β-glucanase activity, and calcium-dependent phospholipid binding, among other MF terms, were uniquely enriched in the up-regulated dataset. However, in the down-regulated dataset, MF terms related to oxidoreductase activity (e.g., D-arabinono-1,4-lactone oxidase, alcohol dehydrogenase (NADP+), glycerol dehydrogenase [NADP+] activity), shikimate kinase activity (e.g., 3-dehydroquinate dehydratase, 3-phosphoshikimate 1-carboxyvinyltransferase activity), and transmembrane transporter activity were enriched.

### 2.8. Different Expression Profile of Fungal Genes during the Colonization of the Host Pines

Dual RNA-Seq analysis has identified several fungal transcripts that were DE during the colonization of pine tree species by *F. circinatum* (Table S14). The EffectorP 2.0 software identified 29 predicted fungal effector-encoding genes (Table S15). In particular, 12 effector-encoding genes (including chaperone-like protein, methyltransferase, oxidoreductase, and pectate lyase C) were significantly up-regulated in *P. pinea* with respect to *P. radiata*. In the down-regulated dataset, 17 effector-encoding genes were identified, including a glycosyl hydrolase, a shikimate 5-dehydrogenase, gluconate-5-dehydrogenase, and a homolog to necrosis-inducing protein. The predicted fungal effector-encoding genes were evaluated for their role in pathogenesis using the PHI-base (Table S6): only two glycosyl hydrolases were assigned as “unaffected pathogenicity” among the down-regulated fungal genes; and two possible effectors were found in the up-regulated dataset, basing on the assumption of the knockout mutants.

Other potential pathogenicity and virulence genes from the PHI-base were found in the DEGs of *F. circinatum* (Table S6). Among the up-regulated genes, 25 were found in this database, and knocking out 10 of these genes in other pathogens has led to mutants of reduced virulence or absence of pathogenicity. Among these, xylanase (endo-1,4-beta-xylanase), protease (MCA1), hydrolase (triacylglycerol lipase V precursor), a cytochrome involved in sterol biosynthesis (CBR1), and a couple of ATPases were present. Five genes potentially associated with virulence were identified in the down-regulated dataset. These were two ammonium permeases (mepA), one gene with glutaminase activity (anthranilate synthase component 2), a GABA-transaminase (4-aminobutyrate aminotransferase), and an oxidoreductase (monoamine oxidase N).

In the *F. circinatum* DEGs dataset, some genes with a predicted role on the degradation of plant cell wall polymers such as cellulose, hemicellulose, and lignin were identified. Several glycoside hydrolases (GH) were DE in both up- and down-regulated datasets. Among the down-regulated genes, one chitinase and one expansin-like protein were identified. A carboxylesterase that belongs to the carbohydrate esterases (CE) family, a lytic polysaccharide monooxygenase (LPMO), an arabinan endo-1,5-alpha-L-arabinosidase and a peptidase M50B-like were up-regulated. Additionally, genes encoding a laccase, a GMC oxidoreductase, and a cellobiose dehydrogenase were also up-regulated.

Transporter encoding genes were mostly down-regulated in *P. pinea* in relation to *P. radiata*. Two amino acid permeases, the major nitrogen transporters, were up-regulated, and three were down-regulated, while other nitrogen transporters such as ammonium and peptide transporters were down-regulated. Two proteases, which help to make plant amino acids available for fungal growth [51], were also DE: aspartic protease was up-regulated, while a serine protease precursor was down-regulated. The highest down-regulated transporter (FC072V.6542.1, close to 10-log_2_ [fold change]) is involved in choline transport. One major facilitator superfamily (MFS) transporter was down-regulated, and other MFS associated with sugar transport was up-regulated during infection. One ABC transporter was down-regulated while other ABC transporters associated with multidrug resistance (MDR) was up-regulated.

Few genes involved in the secondary metabolism, such as shikimate-5-dehydrogenase and dehydroshikimate dehydratase enzymes, were down-regulated. Additionally, a terpene synthase was also down-regulated. The results show an oxidoreductase activity in the pathogen, including a couple of genes encoding oxidoreductase FAD (Flavin Adenine Dinucleotide) protein in the *P. pinea* down-regulated genes. Moreover, several genes that encode enzymes in fatty acid β-oxidation were up- and down-regulated.

## 3. Discussion

The study of the pathosystem involving the resistant *P. pinea* or the susceptible *P. radiata* and the pathogenic fungus *F. circinatum* has provided a wide picture of the transcriptional changes associated with these phenotypes. The use of dual RNA-Seq has allowed evaluating simultaneously the differences in transcript expression of *F. circinatum* when infecting each of the pine species and the respective species-specific response. The availability of *P. taeda* and *F. circinatum* genomes allowed us to map and distinguish between plant and fungal RNAs. As expected, fungal RNA contributed to a low percentage of reads [41], with an average of 1.36%, and out of these, 147 genes were considered differentially expressed. Noticeably, a very low number of fungal reads (0.03%) were detected in mock-inoculated samples, suggesting the presence of endophytic fungi or of conserved eukaryotic sequences.

In previous studies, a marked trend has been observed in the increase in DEGs as the disease was progressing [29,38,42,45]. The transcriptomic comparison of the present study at an early stage of disease (4 dpi) showed that the number of DEGs in *P. pinea* (1822) was vastly higher than in *P. radiata* (371). Assuming that the number of DEGs is correlated with the phase of the host response to the infection, it could be hypothesized that *P. radiata* presents a delayed induction of defense activation. By contrast, all the categories related to host signaling perception and defense response, including lignification, secondary metabolite biosynthesis, and detoxification, were highly induced in the resistant *P. pinea*. However, a degree of overlap exists between the resistant and susceptible host with regard to the enriched GO terms by the defense response mechanisms, suggesting a similar transcriptional change by *F. circinatum* infection. Several examples in forest tree pathosystems have shown a delay in the defense response in susceptible interactions [34,52,53,54], including a *Pinus*-*F. circinatum* study [45]. The early activation of plant defense-related genes requires a straight recognition of pathogen elicitors. PR receptors such as RLKs and receptor-like proteins (RLPs) are relevant players of this perception, as well as the pathogen-specific response by R genes [55,56]. Among the DEGs in *P. pinea*, a large number of PRR containing domains such as LRR, lysine motifs (LysM), and lectin domain were mainly up-regulated. The fact that some of these receptors were down-regulated in *P. pinea* could be related to a blockage by the pathogen effector proteins [56]. Furthermore, two PP2A proteins, which act as negative regulators of plant defense responses [57], were down-regulated in *P. pinea*. In turn, several R genes were mainly up-regulated in this resistant species. On the contrary, the near absence of PRR induction in *P. radiata* and the lack of up-regulation of MAPK and CDPK transcripts could explain the weak downstream defense signaling. In several plant species, MAPK cascade components have been implicated in local resistance signaling and signal transduction mechanisms (see [52] and references therein). Moreover, the presumably interrupted or delayed induction of MATE transporters in *P. radiata*, which play a key role in the timely delivery of antimicrobial metabolites to deter pathogen progression [53], could also be considered as a susceptibility factor in this species.

ROS participates in defense response signaling, however, its excess may damage biomolecules and disrupt plant metabolism. Although some antioxidant systems were excluded from being part of *Pinus* defense against *F. circinatum* (e.g., ascorbate-glutathione cycle) [46], ascorbate seems to have an important role in our experimental system as genes related to L-ascorbate biosynthesis were only up-regulated in *P. pinea*. Concurrent with this, the production of antioxidant secondary metabolites (terpenoids) was represented with a high number of up-regulated terpene synthases in *P. pinea*. An early plant response through the induction of the terpenoid pathway along with ABA biosynthesis has been previously observed in *Linum usitatissimum* infected by *Fusarium oxysporum* [58]. On the other hand, ABA signaling seems to have a role in the *F. circinatum* infection. While two protein kinases, CIPK20 were up-regulated in *P. pinea*, the enzyme responsible for ABA catabolism into the less active phaseic acid (PA) was induced in *P. radiata*. It has been proved that PA has some ABA-like activity and is able to activate some ABA receptors [59]. The inactive form of ABA (ABA-GE), which is usually involved in ABA transport or accumulation, is transformed to its active form under stress by β-glucosidases [60]. The genes that encode these enzymes were highly induced in both pine species.

In this study, GO terms related to JA and ET pathways were over-represented in both pine species. Genes participating in JA biosynthesis such as OPR were up-regulated during pathogenesis, although to less extent in *P. radiata*. However, JA was not accumulated in the needles of any of these species at the early stages of PPC disease [46]. Nonetheless, the activation of root OPR genes was delayed in an apple tree genotype susceptible to *Pythium ultimum* infection [53], which could also be the case for the susceptible *P. radiata* in response to *F. circinatum*. In addition, *F. circinatum* infection could have activated the JMT genes that catalyze the formation of methyl jasmonate (MeJA) from JA in *P. pinea* and *P. radiata*. Although the pre-treatment of MeJA was unsuccessful in protection against *F. circinatum* in different *Pinus* species [61,62], this component could play an important role in the induction of other response defenses. MeJA-mediated cellular responses include the induction of genes encoding terpene synthases [63] and enzymes of the JA biosynthesis such as LOX [64], both widely up-regulated in the two pine species. Although these results could indicate an active biosynthesis of JA mainly in *P. pinea*, the down-regulation of COI1 together with the high induction of JAZ genes could denote a suppression of JA signaling, as observed in *P. pinaster* infected with *F. circinatum* at 5 and 10 dpi [42]. Our results further support that *F. circinatum* may target and block JA signaling by COI1 suppression as hypothesized by Hernandez-Escribano et al. [42].

The induction of genes encoding enzymes responsible for ET biosynthesis and the inhibition of the copper transporter RAN1, whose lack of expression leads to the constitutive expression of ethylene response [65], indicate a coordinated role of ET in *P. pinea* defense. In this study, only one ethylene response factor (ERF6), which integrates signals from ET and JA pathways playing a role in the regulation of plant defense responses, was up-regulated in both pine species. The expression of this transcription factor is induced very rapidly and regulates both stress tolerance and growth inhibition [66], playing a positive role in *Arabidiopsis* defense against *Botrytis cinerea* [67]. Additionally, ERFs may be involved in the suppression of SA-mediated signaling and SA responsive gene PR1. Although *P. radiata* presented a 12-fold change of PR1 gene expression, two PAD4 were down-regulated in this species, which could reflect the antagonism of SA and JA/ET pathways. On the other hand, the results showed a moderate activation of SA signaling in *P. pinea* together with the high up-regulation of two PR1 genes. Induction of PR1 genes upon *F. circinatum* infection has also been observed in *P. pinaster* and in the resistant genotype of *P. radiata* [38,42]. In addition, the synergistic cooperation of SA and JA has been extensively reported [42,45,68].

Amaral et al. [46] showed that while the susceptible *P. radiata* suffered photosynthesis impairment once PPC symptoms occur, *P. pinea* was able to maintain its photosynthetic activity over time after *F. circinatum* inoculation. At the transcriptional level, the down-regulation of genes encoding RuBisCo, photosystem I reaction center subunit or chlorophyll a/b binding protein (Table S19), due possibly to the higher levels of ethylene [69], could suggest a reduction in photosynthesis activity in *P. pinea*. However, this was not reflected in the physiological analysis results. On the other hand, the photosynthesis in *P. radiata* was altered neither at the transcriptional level nor in the physiological analysis. This highlights the importance of an integrated study of transcriptomic and physiological analysis that considers the post-transcriptional regulation. The general stomata opening and increased transpiration rate verified in *P. pinea* upon inoculation with *F. circinatum* in Amaral et al. [46] are at odds with our results. The absence of these changes in our study may be explained by the different plant growing conditions or plant provenances.

A large number of predicted genes related to cell wall reinforcement and lignification were induced in the resistant species, together with a strong regulation of cell wall modification through inhibition of all expressed cellulose synthase proteins, expansins, and some xyloglucan endo-transglycosylase (XET) proteins. The suppression of cellulose synthesis has been previously associated with enhanced resistance to fungal and bacterial pathogens [70]. Likewise, the down-regulation of expansins strengthens the cell wall by avoiding the loosening of the wall by cell extension. Interestingly, a predicted hydroxyproline-rich glycoprotein (HRGP) was only up-regulated in *P. pinea*, being these structural proteins induced in disease-resistant responses [71]. The fact that some genes predicted to encode XET proteins were down-regulated could be related to the GO terms enriched in the up-regulated DEGs of *F. circinatum* that were associated with the degradation of the cell wall components such as polysaccharide, hemicellulose, and xyloglucan. In fact, it has been suggested that pathogens could target and inhibit cell wall repairing enzymes such as XET to achieve colonization of the host tissue [72]. On the other hand, pectinesterases, whose degree of methylesterification determines cell wall solidity and cooperates to its disassembly [73], were down-regulated in *P. pinea*, with their inhibitors PMEI being the most induced genes in this species after *F. circinatum* infection. In contrast, one PMEI was up-regulated, but another one was down-regulated in *P. radiata* that would result in increased exposure to pathogen infection in this species. PMEI activity has been associated with resistant genotypes in several plant species [73], and our results indicate its role also in the resistance to *F. circinatum*.

Another structural defense induced in conifers is early fibers lignification to arrest hyphal penetration at the site of infection. Previous studies of plants challenged with pathogens have shown the induction of genes involved in lignin biosynthesis [29,30,31,37]. In *P. pinea*, the main enzymes involved in the lignin biosynthesis were induced by *F. circinatum* infection, including two dirigent-like disease resistance predicted proteins that are associated with lignin biosynthesis and response to pathogens [74]. Noteworthy, the gene that encodes the phenylalanine ammonia-lyase (PAL), the enzyme that leads to lignin biosynthesis, was not present in the DEGs of *P. radiata* in spite of its key role in linking plant primary and secondary metabolism. These results suggest a quick *P. pinea* response in the reinforcement of cell walls by lignification, contrasting with the weak regulation of these genes in *P. radiata*.

Besides branching into lignin, the phenylpropanoid pathway leads to flavonoid/isoflavonoid biosynthesis [75]. Starting with the up-regulation of the gene encoding PAL enzyme, the resistant species induced an active and wide defense reaction covering almost completely the flavonoid pathway (Figure 7), which includes the induction of a large number of CHS1 (the first enzyme of this pathway) and CHI (isomerize chalcone to flavanone) [76] transcripts. Recent studies have reported an important role of flavonoid pathway components in the resistance of *Picea abies* to *Heterobasidion* infection [37,77,78], being one of the main induced pathways in asymptomatic trees. The moderate resistant *P. pinaster* challenged with *F. circinatum* showed that the induction of flavonoid biosynthesis was maintained over time (until 10 dpi) [42]. In accordance, although chalcone synthesis was highly induced in *P. radiata* as well, the downstream components of this pathway were poorly represented. As flavonoids have antioxidant properties [79], the oxidative stress and the subsequent induction of SA caused by the *F. circinatum* infection could be attenuated by the early response of flavonoid compounds and GSH/GST proteins in *P. pinea*. In line with this, the most overexpressed GO terms and KEGG pathways in *P. pinea* were related to phenylpropanoid, chalcone, flavonoid, anthocyanin, and secondary metabolite synthesis. Noteworthy, both species showed induction of LAR proteins upon pathogen infection, which suggests the production of proanthocyanidins as a response to *F. circinatum* infection and alkaloid berberine proteins that inhibit the multiplication of fungi [80].

Under stress, PR proteins are indispensable for plant immune responses [81], conferring local or systemic resistance. In addition to PR1 proteins, a large number of genes with antimicrobial properties that encode other PR proteins, including PR2, PR3, PR5, PR9, PR10, and PR14, were mainly up-regulated. This is in accordance with several studies on forest tree-pathogen interaction [19,27,29,30,32,36,38,40,42,82]. It has been reported that, after *F. circinatum* infection, JA and SA induce chitinases (PR3) [83] that degrade chitin, a major component of fungal cell walls. The presence of β-1,3-endoglucanases (PR2) greatly enhances the antifungal properties of PR3 by degrading the glucan matrix in which chitin is embedded [84]. Several genes encoding PR3 proteins were up-regulated in both pine species; however, a PR2 protein was only induced in *P. pinea*, suggesting a less effective response of *P. radiata* at an early stage of the disease. Likewise, the plant PR5 family, known as thaumatin-like protein (TLP), has also shown antifungal properties against several forest tree pathogens [27,29,37,54,85]. Additionally, Carrasco et al. [38] suggested the possible role of PR1 and PR5 in the activation of the SA-dependent pathway, resulting in the induction of systemic induced resistance (SIR) against PPC in *P. radiata*. Other PR proteins such as peroxidases (PR9) were up- and down-regulated in *P. pinea*, similar to the interaction of *P. pinaster*-*F. circinatum* at 10 dpi [42] and *Eucalyptus nitens*-*Phytophthora cinnamomi* at 5 dpi [36]. This could point out pine PR9 as effector target of *F. circinatum*, which has been found in several *Phytophthora* species infecting *Carica papaya* [86,87,88]. It has been suggested that activation of PR genes, notably plant chitinases, is produced mainly in susceptible *Pinus* spp. during PPC disease [19,83,89,90]. However, these studies have remarked that in resistant plants, the highest expression levels of these genes are detected in early stages (2–3 dpi), decreasing over time, while the opposite occurs in susceptible plants where an accumulation of these transcripts can be noted at 8–14 dpi. Our results support the previous conclusions since *P. pinea* has shown a higher number of induced PR genes than *P. radiata* at 4 dpi.

Among conifers, one of the most common induced defenses against pathogens is an early lignification of fibers [91]. In the up-regulated genes of the pathogen when infecting *P. pinea*, the most representative GO terms were related to the degradation of the plant cell wall. Indeed, *F. circinatum* transcripts matching genes that encode for cell wall-degrading enzymes (CWDE) were detected in greater numbers in the up-regulated dataset. Several glycoside hydrolases were identified, which are responsible for the hydrolysis of the sugar residues link in cellulose and hemicelluloses [92]. Among these, a gene involved in the conversion of a plant cell wall polysaccharides into fermentable sugars, the arabinan endo-1,5-alpha-L-arabinosidase, was also detected. In addition, genes with a predicted role in lignin degradation, such as laccases, GMC oxidoreductases, and cellobiose dehydrogenases, were uniquely present in the up-regulated gene group. This could suggest a differential activity of *F. circinatum* in the degradation of lignin between *P. pinea* and *P. radiata*, which could be associated with the lignin content in each species. Moreover, the strong reaction in cell wall reinforcement observed in *P. pinea* may be behind the activation of several CWDE by the pathogen. Additionally, several terms related to ornithine metabolism were enriched in the up-regulated genes. Ornithine is a non-protein amino acid that participates in the plant response to stress, which could indicate sequestration of potential plant defenses for the nutritional benefit of the pathogen during invasion. On the other hand, enzymes such as the expansin-like protein that induces extensibility and stress relaxation of plant cell walls [93] were identified as down-regulated in the pathogen infecting *P. pinea* in comparison to *P. radiata*. In addition, fungal chitinases that are presumably involved in fungal cell wall remodelings, such as spore germination or hyphal tip growth [94], were also identified in this dataset.

The first strategy of pathogens to obtain nutrients from the plant involves the use of transporters, and their ability to use those nutrient resources determines as much the success of the invasion [95,96]. The functional analysis revealed a noticeable enrichment of nutrient transport in the *P. pinea* down-regulated genes, especially of nitrogen sources including ammonium and amino acids. Genes such as amino acid permeases (e.g., cholin permease and carnitine transporter), alcohol dehydrogenases (s-(hydroxymethyl) glutathione dehydrogenase alcohol dehydrogenase), or formamidases were present in this group, being therefore over-represented when infecting *P. radiata*. Furthermore, choline transport, known as the promoter of *Fusarium graminearum* growth and virulence [97], was highly down-regulated in *P. pinea* in comparison to *P. radiata*. Interestingly, several nodulin-like proteins were highly induced in *P. radiata*. The induction of these proteins in plant-microbe interaction has been associated with enhancing pathogen fitness by its control over plant transporters [98]. The large-scale induction of transporters for the uptake of nutrients has been associated with the shift from the biotrophic to necrotrophic phase in hemibiotrophic pathogens due to the rapid growth of secondary hyphae in the latter phase [99]. Likewise, genes encoding proteins with roles in nutrient transport were highly up-regulated at the early stages of *F. oxysporum* colonization of susceptible seedlings of *Medicago truncatula* [100]. It has been demonstrated that the nitrogen availability in fungi modulates its growth, differentiation, and the biosynthesis of many secondary metabolites [101]; accordingly, pathogens will cause less disease in plants where nitrogen is limiting [102]. Moreover, a general accumulation of amino acids, conceivably associated with plant stress response and/or pathogen hijacking of host metabolism, was reported in the susceptible *P. radiata* upon *F. circinatum* inoculation [19]. Therefore, the enrichment of genes related to the uptake of nitrogen by *F. circinatum* infecting *P. radiata* could provide the pathogen a competitive advantage in the plant-pathogen interaction.

## 4. Materials and Methods

### 4.1. Fungal Isolate, Plant Material, and Inoculation Trial

The *F. circinatum* isolate (Fc072v) used in this work belongs to mating type 2 (MAT-2) and was isolated from an infected *P. radiata* tree located in the North of Spain (Cantabria, Spain). Plant material consisted of one-year-old seedlings of *P. radiata* (Provenance: Galicia, Spain) and *P. pinea* (Provenance: Meseta Norte, Spain).

The spore suspension was obtained from *F. circinatum* cultured on PDB medium (2.40% *w*/*v* potato dextrose broth, Scharlab S.L., Barcelona, Spain). For that, an Erlenmeyer flask containing 1 L of PDB and 5 mycelial agar plugs (diameter 4–5 mm) obtained from the margin of an actively growing colony was placed on an orbital shaker at 140 cycles for 48 h at 25 ºC. Finally, the spore suspension was obtained by filtering twice through sterile cheesecloth to remove hyphae and was adjusted with a hemocytometer at 10^6^ spores mL^−1^.

Pathogenicity tests were carried out by the stem inoculation technique [7]. Briefly, a wound was made with a sterile scalpel 5–7 cm above the collar of each plant after the removal of needles from that area. Then 24 seedlings of each species in full growth were inoculated with 10 μL of the spore suspension, and another 24 control seedlings were mock-inoculated in the same way with sterilized distilled water. The inoculated wound was immediately sealed with Parafilm^®^ to prevent drying, and the seedlings were placed in a growth chamber at 21.5 °C, with a 16/8 h light/dark photoperiod. Watering and other procedures were conducted as per routine nursery practice, except that no fertilizers or fungicides were applied.

Each biological replicate was represented by an individual seedling in this study. Six biological replicates per treatment were used for physiological parameters, except for the condition of *P. pinea* inoculated by *F. circinatum* with four biological replicates. On the other hand, four biological replicates per treatment were used for the gene expression analysis. Sampling for transcriptomic analysis and physiological measurements occurred 4 days after inoculation (dpi). The remaining seedlings were used to check the susceptibility of each species to *F. circinatum*. In particular, seedling mortality was estimated twice a week, and survival analysis based on the nonparametric estimator Kaplan–Meier [103] was performed with the “Survival” package [104] to test the mortality up to the end of the experiment (84 days). Survival curves were created with the “Survfit” function, and the differences between the curves were tested with the “Survdiff” function. All analyses were performed using R software environment [105].

### 4.2. Water Potential and Needle Gas Exchange-Related Parameters

Midday stem water potential (Ψ_md_, MPa) was measured for every seedling using a Scholander-type pressure chamber (PMS Instrument Co., Albany, OR, USA). The apical shoot net CO_2_ assimilation rate (A, µmol CO2 m^−2^·s^−1^), transpiration rate (E, mmol H_2_O m^−2^·s^−1^), stomatal conductance (gs, mol H_2_O m^−2^·s^−1^) and sub-stomatal CO_2_ concentration (Ci, vpm) were measured using an infra-red gas exchange analyzer (LCpro-SD, ADC BioScientific Limited, Hoddesdon, U.K.) with a conifer-type chamber.

The Shapiro–Wilk’s and Bartlett’s tests were used to test for normality and homoscedasticity of the data (*p* ≤ 0.05). A two-way analysis of variance (ANOVA) was performed on each physiological parameter in order to evaluate the *F. circinatum* and species effect. When data did not follow ANOVA’s assumptions, robust statistical methods were applied [106]. Particularly, heteroscedastic two-way ANOVAs were carried out using the generalized Welch procedure and a 0.1 trimmed mean transformation. ANOVAs were carried out using the “Wilcox’’ Robust Statistics (WRS2)” package, with the functions “t3way” and “lincon” [107] using R software [107]. Data are presented as mean ± SE (standard error).

### 4.3. RNA Extraction, Library Preparation, and Sequencing

Total RNA extractions were performed from 100 mg of stem at the inoculation point of each sample using the method described by Valledor et al. [108]. The total RNAs were submitted to Macrogen (Macrogen, Korea) for sequencing. Paired-end libraries with fragments of 150 bp were prepared using poly(A) selection with the TruSeq Stranded mRNA LT sample preparation Kit (Illumina, San Diego, CA, USA) for a coverage depth of 80 M reads. Subsequently, libraries were sequenced by Illumina NovaSeq 6000 platform.

### 4.4. Pre-Processing of Raw Data and Mapping of Reads

Raw reads have been deposited in the NCBI SRA Database under accession numbers SRR13737940-53 (BioProject PRJNA702546). All RNA sequence files were first assessed for quality control using FastQC v.0.11.9 [109]. The raw reads were trimmed for Illumina adaptor sequences and low-quality base-calls using Trimmomatic v.0.38 [110].

To perform the reference-based alignment for the host, both pine species (*P. radiata* and *P. pinea*) were treated equally using the assembled *Pinus taeda* genome (Pita_v2.01; Treegenes database [111]). Reads were mapped to the *P. taeda* genome with HiSat2 v.2.0.0 [112] with the default settings to obtain SAM files. The files containing information of the mapped pine reads were then analyzed with FeatureCounts v.1.4.0 (Subread package) [113] to obtain read counts for gene regions specified in a corresponding GTF file (Pita_v2.01; Treegenes database [111]) for the *P. taeda* genome sequence. The resulting count files were then manually formatted into a count matrix suitable for differential expression (DE) analysis.

For the pathogen, the sequenced genome of the isolate Fc072v of *F. circinatum* was used for alignment with HiSat2 v.2.0.0. with the default settings to obtain SAM files. Due to the lack of available annotation files for this pathogenic fungus, a transcriptome assembly was conducted. Before assembly, every SAM file was piped to SAMtools utility for generating an alignment file in binary alignment map (BAM) format and sorting [114]. StringTie v.2.1.4 was then used to assemble the transcriptome using the BAM file from each sample, merging all assemblies into an experiment-level reference assembly and estimating the abundances of all transcripts assembled, mapping again the reads to the experiment-level reference [115]. The output file was reformatted for further analysis in edgeR using the “prepDE.py” script available at https://ccb.jhu.edu/software/stringtie/index.shtml?t=manual (accessed on 15 April 2020).

### 4.5. Annotation

The *F. circinatum* experiment-level reference assembly was converted to fasta format using Gffread v.0.12.1 [116]. The output file was annotated with EnTAP v.0.9.2 [117] as follows. The pipeline started using GeneMarkS-T v.5.1 [118] for open reading frame prediction. After that, the similarity search was conducted by BLASTp using the NBCI non-redundant protein database (release-201), RefSeq complete protein database (release-201), and the UniProtKB/Swissprot database (release-2020_05) through DIAMOND v.1.9.2 [119] with default settings. The orthologous group assignment to gene families including protein domains (SMART/Pfam), gene ontology (GO) terms, and KEGG pathway was performed with EggNOG v.1.0.3 [120] and InterProScan v.5.47–82.0 [121]. The predicted *F. circinatum* proteins were also used to investigate whether they had been verified to be pathogenic genes using annotations from the Pathogen–Host Interaction database (PHI-base) [122] using BLASTp (*e*-value <  0.00001). In addition, the machine learning predictor EffectorP [123] trained for fungal secreted proteins were used to identify potential effectors among the differentially expressed genes of *F. circinatum*. The functional analysis of the host transcripts was performed used the updated annotation file of *P. taeda* (Pita.2_01.entap_annotations.tsv; Treegenes database [111]). The file was manually adjusted for further analysis.

### 4.6. Transcript Expression Analysis

The counts matrix tables were loaded into the software R v.3.6.2 [105], and both pine and fungal RNA-Seq data were analyzed using edgeR v.1.3.959 package [124], performing the following steps. First, data sets were normalized in order to remove non-biological variation and to make values comparable across the samples. Normalization of read counts was conducted with the trimmed mean of M-values (TMM) method of edgeR. Afterward, each transcript was fitted to a generalized linear model following a negative binomial distribution, and statistical testing for the differential gene expression (DGE) significance was performed using the empirical Bayes quasi-likelihood F-tests. Computed *p*-values were adjusted using the false discovery rate (FDR) of Benjamini-Hochberg to control for multiple testing [125]. Pairwise comparisons for the DE of pine transcripts were performed for inoculated vs. mock-inoculated. For *F. circinatum* transcripts, a pairwise comparison between *P. pinea* vs. *P. radiata* conditions was conducted. The identification of differential expression genes (DEGs) was determined using the threshold of log_2_ (|Fold-change|) ≥ 1.5 at a false discovery rate of (FDR) lower than 0.05. To visualize the similarity of the replicates and identify any sample outliers, the principal component analysis (PCA) was performed using the log_2_ fold changes for the host and the pathogen datasets.

### 4.7. Functional Analysis

In order to perform an efficient functional analysis, the DEGs of each dataset were divided into up- and down-regulated subsets [126]. Using all genes as background, GO and KEGG enrichment analysis of the DEGs were implemented by GOSeq v.1.38.0 based on the Wallenius non-central hyper-geometric distribution that allows the adjustment for DEGs length bias [127]. These analyses were carried out for the three different comparisons (inoculated vs. mock-inoculated for both pine species and pathogen gene expression comparisons). GO terms were considered significantly enriched if the *p*-value was lower than 0.05.

## 5. Conclusions

In this article, a comprehensive transcriptional study of the host-pathogen interaction between the hemibiotrophic pathogen *F. circinatum* and a resistant and a susceptible host species is presented. The lack of a substantial response in *P. radiata* contrasted with an advanced transcriptional reprogramming for defense in *P. pinea* at an early stage of the disease. The results have suggested that the weak response of *P. radiata* could be related to the impaired perception of the fungal infection since early defense responses, including calcium flux, recognition by R proteins, or the activation of mitogen-activated protein kinases (MAPKs), were absent in this species. The failure during the initial infection stage that in turn are involved in signaling the intermediate and late responses has presumably led to a weaker activation of a diverse array of defense pathways, including lignification, phytohormone biosynthesis, and the production of non-enzymatic antioxidants such as ascorbate and flavonoids. This circumstance may enable the fungus to take full advantage of *P. radiata* nutrients (such as nitrogen), as suggested by the transcripts analysis of *F. circinatum*. Our findings allow a better understanding of the pine-*F. circinatum* interaction and of conifer defense responses to biotic stress and set the foundation for future studies for validating the association of these candidate genes with PPC resistance traits. This knowledge will be implemented in the breeding programs for the commercial deployment of resistant pine reproductive material.

## Figures and Tables

**Table 1:** Summary of differentially expressed genes identified in *P. pinea* and *P. radiata* for each comparison.

Organism	Genes Up-Regulated ^1^	Genes Down-Regulated ^1^
Differentially expressed host genes ^2^		
*P. pinea*	1307	515
*P. radiata*	359	12
Differentially expressed pathogen genes ^3^		
*F. circinatum*	118	86

^1^ Number of up-regulated and down-regulated significantly differentially expressed genes (false discovery rate, FDR < 0.05 and a log_2_(fold change) > 0.5 or < −0.5) using the QLTest (Benjamini and Hochberg FDR correction) with edgeR. ^2^ Host genes differentially expressed in inoculated relative to mock-inoculated host expression data. ^3^ Differentially expressed *F. circinatum* genes in *P. pinea* relative to *P. radiata* inoculated samples from pathogen expression data.

## Data Availability

Transcriptome data are available at NCBI database (PRJNA702546).

## Supplementary Materials

The following are available online at https://www.mdpi.com/article/10.3390/ijms22105231/s1, Table S1: Read mapping statistics to the host (*Pinus taeda*) and the pathogen (*Fusarium circinatum*) reference genomes, Table S2: List of differentially expressed *P. pinea* genes, Table S3: List of differentially expressed *P. radiata* genes, Table S4: List of differentially expressed genes of *Fusarium circinatum*, Table S5: Final result of the EnTAP annotation for the *Fusarium circinatum* transcriptome, Table S6: PHI-base alignments for the *Fusarium circinatum* transcriptome, Table S7: Significantly enriched GO terms identified from differentially expressed *P. pinea* genes, Table S8: Significantly enriched KEGG pathways identified from differentially expressed *P. pinea* genes, Table S9: Significantly enriched GO terms identified from differentially expressed *P. radiata* genes, Table S10: Significantly enriched KEGG pathways identified from differentially expressed *P. radiata* genes, Table S11: Genes involved in plant defense with differential expression (log2) in *Pinus pinea* and *P. radiata* following challenge with *Fusarium circinatum*, Table S12: Phytohormone related DEGs in the hosts, Table S13: Significantly enriched GO terms identified from differentially expressed genes in *F. circinatum*, Table S14: Fungal transcripts related to pathogenesis expressed during the colonization, Table S15: Alignments for the differentially expressed genes of *Fusarium circinatum* to the Machine Learning algoritm for the prediction of fungal effectors, Figure S1: Water potential of *P. pinea* and *P. radiata* inoculated with *F. circinatum* (dark gray) and controls (light gray). Error bars show the standard deviation. Different letters indicate significant differences (ANOVA, *p* < 0.05), Figure S2: Two-dimensional scatterplot of the principal component analyses (PCA) for *Pinus radiata* and *P. pinea* where distances approximate the typical log2 fold changes between the samples. Red arrows represent indicate the discarded samples for the downstream analysis, Figure S3: Differential gene expression was analyzed using the “EdgeR” R package and plotted as a MA plot. The log2 fold change of the normalized gene expression between inoculated and control treatments of both pine species is represented on the y-axis, and the average log counts per million (CPM) are plotted on the x-axis. Color dots represent the differentially expressed genes: red dots indicate up-regulated genes, and blue dots down-regulated genes. Black small dots represent genes with a similar expression between treatments, Figure S4: Differential gene expression was analyzed using the “EdgeR” R package and plotted as a MA plot. The log2 fold change of the normalized gene expression of *Fusarium circinatum* infecting *Pinus pinea* in comparison with *P. radiata* is represented on the y-axis, and the average log counts per million (CPM) is plotted on the x-axis. Color dots represent the differentially expressed genes: red dots indicate up-regulated genes, and blue dots down-regulated genes. Black small dots represent fungal genes with a similar expression between pine species.
