# Supplementary material for: Dual RNA-Sequencing Analysis of Resistant (Pinus pinea) and Susceptible (Pinus radiata) Hosts during Fusarium circinatum Challenge
Source: Int J Mol Sci. 2021 May 15;22(10):5231. doi: 10.3390/ijms22105231 (PMC8156185; doi:10.3390/ijms22105231)
Supplement: Supplementary file 1 [file ijms-22-05231-s001.zip › Supplementary_Figures.pdf]

Supplementary Material

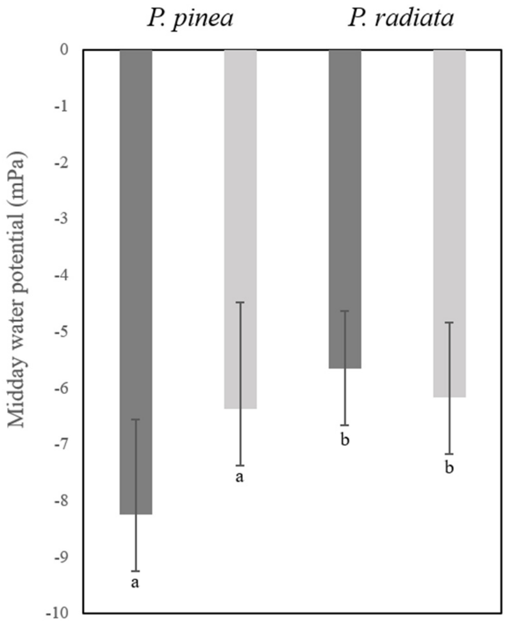

Figure S1. Water potential of *P. pinea* and *P. radiata* inoculated with *F. circinatum* (dark grey) and controls (light grey). Error bars show the standard deviation. Different letters indicate significant differences (ANOVA, $p < 0.05$).

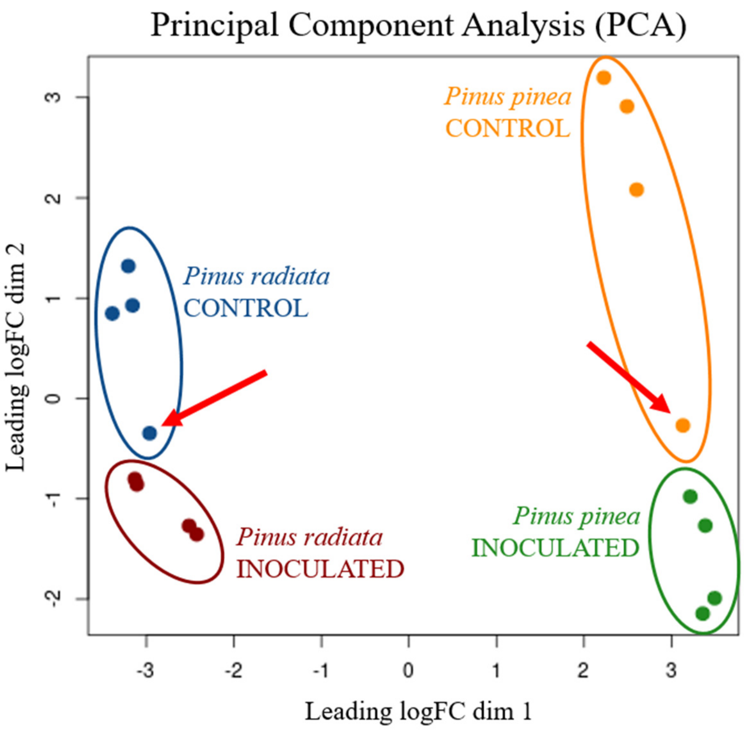

Figure S2. Two-dimensional scatterplot of the principal component analyses (PCA) for *Pinus radiata* and *P. pinea* where distances approximate the typical log2 fold changes between the samples. Red arrows represent indicate the discarded samples for the downstream analysis.

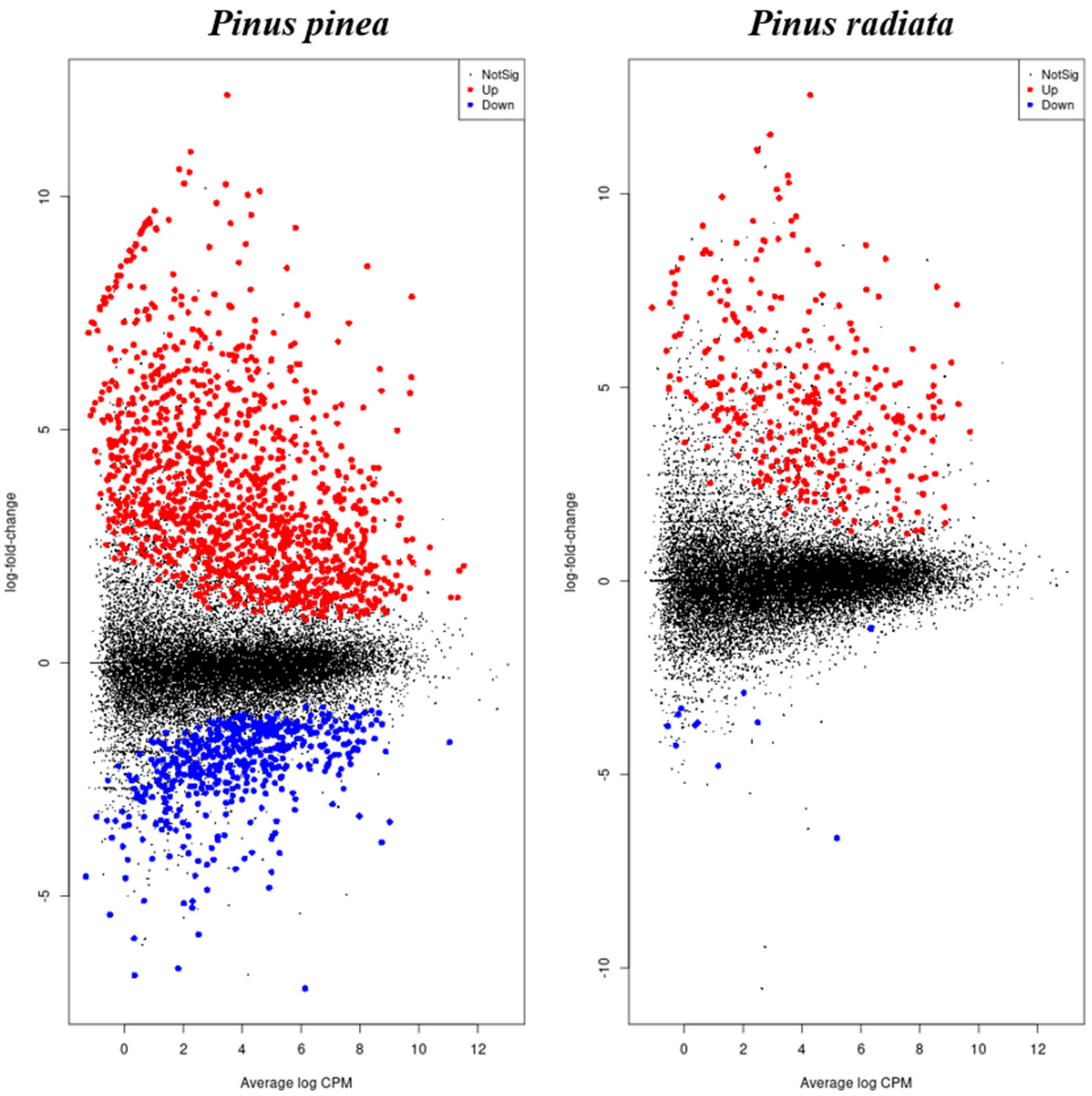

Figure S3. Differential gene expression was analyzed using the "EdgeR" R package and plotted as a MA plot. The log2 fold change of the normalized gene expression between inoculated and control treatments of both pine species, is represented on the y-axis; and the average log counts per million (CPM) is plotted on the x-axis. Color dots represent the differentially expressed genes: red dots indicate up-regulated genes and blue dots down-regulated genes. Black small dots represent genes with similar expression between treatments.

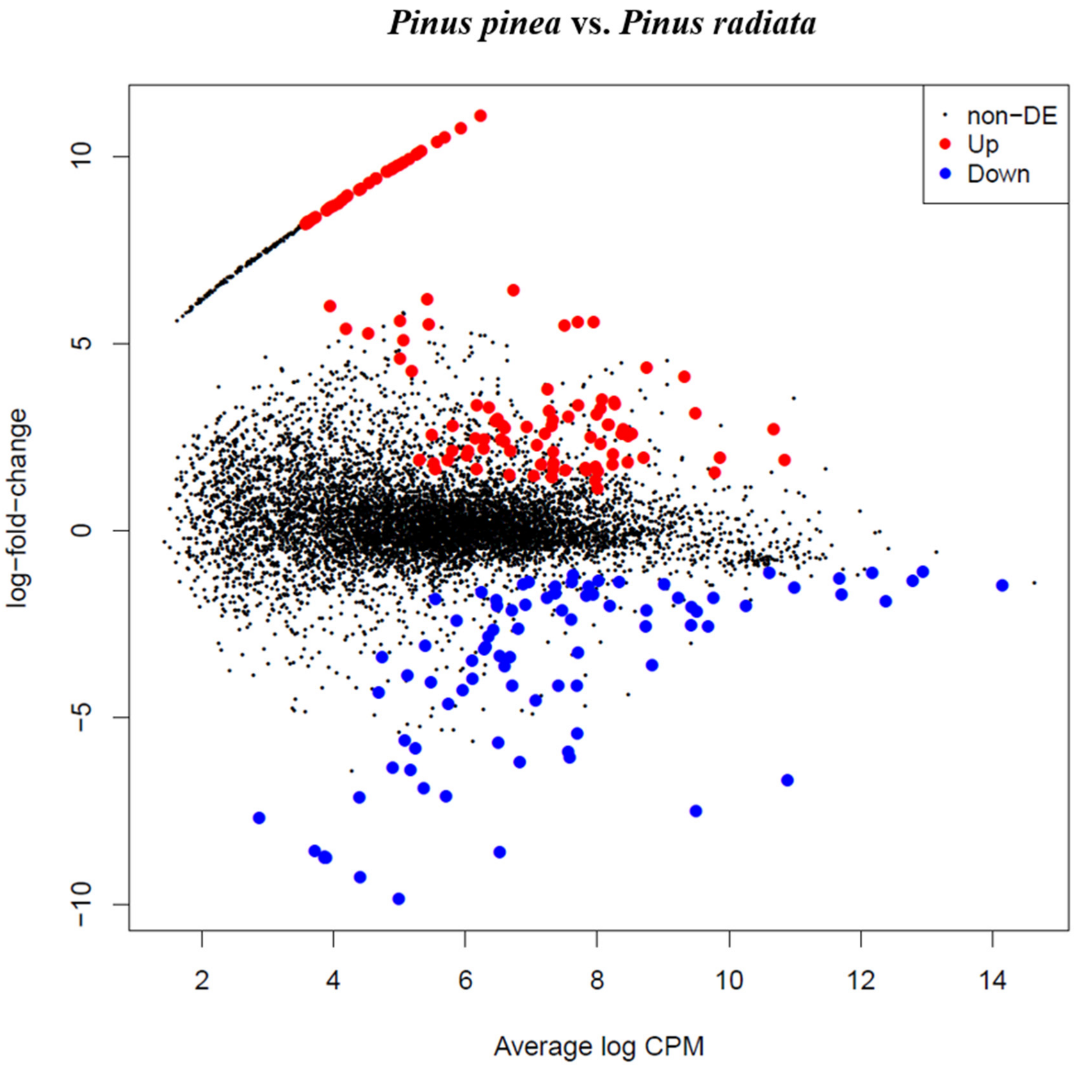

Figure S4. Differential gene expression was analyzed using the "EdgeR" R package and plotted as a MA plot. The log2 fold change of the normalized gene expression of *Fusarium circinatum* infecting *Pinus pinea* in comparison with *P. radiata,* is represented on the y-axis; and the average log counts per million (CPM) is plotted on the x-axis. Color dots represent the differentially expressed genes: red dots indicate up-regulated genes and blue dots down-regulated genes. Black small dots represent fungal genes with similar expression between pine species.
